# Disentangling genotype and environment specific latent features for improved trait prediction using a compositional autoencoder

**DOI:** 10.3389/fpls.2024.1476070

**Published:** 2024-12-16

**Authors:** Anirudha Powadi, Talukder Zaki Jubery, Michael C. Tross, James C. Schnable, Baskar Ganapathysubramanian

**Affiliations:** ^1^ Department of Electrical and Computer Engineering, Iowa State University, Ames, IA, United States; ^2^ Translational AI Research and Education Center, Iowa State University, Ames, IA, United States; ^3^ Department of Agronomy and Horticulture, University of Nebraska-Lincoln, Lincoln, NE, United States; ^4^ Center for Plant Science Innovation, University of Nebraska-Lincoln, Lincoln, NE, United States; ^5^ Department of Mechanical Engineering, Iowa State University, Ames, IA, United States; ^6^ Plant Science Institute, Iowa State University, Ames, IA, United States

**Keywords:** hierarchical disentanglement, latent disentanglement, plant phenotyping, days to pollen, yield, GxE

## Abstract

In plant breeding and genetics, predictive models traditionally rely on compact representations of high-dimensional data, often using methods like Principal Component Analysis (PCA) and, more recently, Autoencoders (AE). However, these methods do not separate genotype-specific and environment-specific features, limiting their ability to accurately predict traits influenced by both genetic and environmental factors. We hypothesize that disentangling these representations into genotype-specific and environment-specific components can enhance predictive models. To test this, we developed a compositional autoencoder (CAE) that decomposes high-dimensional data into distinct genotype-specific and environment-specific latent features. Our CAE framework employed a hierarchical architecture within an autoencoder to effectively separate these entangled latent features. Applied to a maize diversity panel dataset, the CAE demonstrated superior modeling of environmental influences and out-performs PCA (principal component analysis), PLSR (Partial Least square regression) and vanilla autoencoders by 7 times for ‘Days to Pollen’ trait and 10 times improved predictive performance for ‘Yield’. By disentangling latent features, the CAE provided a powerful tool for precision breeding and genetic research. This work has significantly enhanced trait prediction models, advancing agricultural and biological sciences.

## 1 Introduction

Advances in imaging and robotic technologies are making both high-resolution images and sensor data increasingly accessible to plant biologists and breeders as tools to capture measurements of plant traits. These data types can be used to measure or predict traits that are labor-intensive or costly to measure directly, including variation in plant architectural and biochemical traits as well as resistance or susceptibility to specific biotic stresses. A growing body of evidence suggests high dimensional trait datasets can also be useful to predict crop productivity (e.g. grain yield) (Adak et al., 2023; Jin et al., 2024). However, like the plant traits plant biologists and breeders seek to predict, sensor data and the high dimensional traits extracted from that data reflect the impact of both genetic and environmental factors.

Traditionally, such data are analyzed in raw form or by using handcrafted features without explicitly separating genotype (G) and environment (E) factors. Handcrafting features for high-dimensional data can be challenging due to the ‘curse of dimensionality,’ where increasing complexity hinders interpretability, accuracy, and generalizability of models across environments and genotypes. In contrast, latent features derived from unsupervised learning methods capture underlying patterns without the biases of human assumptions, providing more generalizable models for predicting complex traits (Feldmann et al., 2021; Aguate et al., 2017).

Latent phenotyping has emerged as a promising approach to minimize human bias by reducing data dimensionality via unsupervised or self-supervised approaches (Gage et al., 2019; Ubbens et al., 2020; Feldmann et al., 2021; Tross et al., 2023). Traditionally, machine learning methods like PCA (Principal component analysis), Linear Discriminant Analysis (LDA), T-distributed Stochastic Neighbor Embedding (t-SNE), and autoencoders have been used to extract the ‘latent representation’ from high-dimensional data (Alexander et al., 2022; Zhong et al., 2016; Kopf and Claassen, 2021; Song et al., 2023; Gomari et al., 2022; Iwasaki et al., 2023). Autoencoders, in particular, offer advantages in capturing non-linear relationships. By compressing data into a latent space and reconstructing the original input, autoencoders learn a compact yet informative representation crucial for phenotyping (Gage et al., 2019; Ubbens et al., 2020; Tross et al., 2023). Autoencoder-derived representations, though informative, often fail to separate genotype and environment influences, leading to ‘entangled’ latent spaces where distinct plant attributes, such as ‘leaf number,’ ‘height,’ and ‘chlorophyll concentration,’ are intermixed rather than independently represented. Disentangling these attributes within the latent space can improve latent factors’ interpretability.

**Figure 1:**
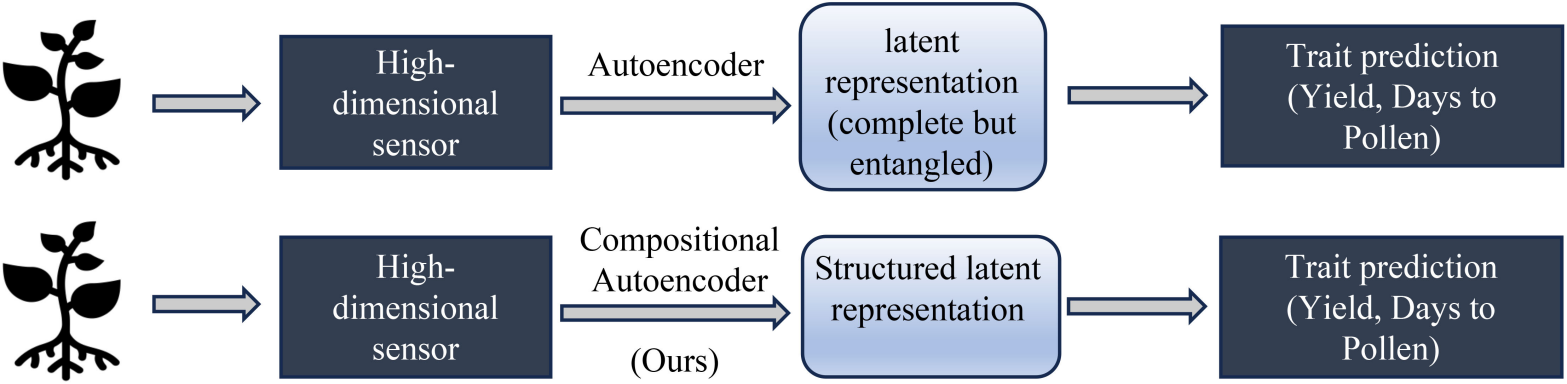
Trait prediction workflow of a Vanilla Autoencoder vs Compositional Autonencoder.

Our hypothesis is that disentangling genotype and environment effects within the latent space can improve prediction accuracy and enhance model generalizability to new genotypes and environments. Specifically, we aim to separate environmental factors (e.g., soil conditions, weather, treatment) and genetic influences in high-dimensional hyperspectral data representing maize phenotypes. We believe that disentangling the latent space into environment and gene effects should help improve the predictive performance of the learned representation on many downstream tasks, as shown in **Figure 1**.

Several disentanglement methods have been proposed, though they often compromise reconstruction accuracy. A common strategy involves regularization techniques, where additional terms in the loss function, as seen in variational autoencoders (VAEs) (Kingma and Welling, 2019), encourage independence among latent variables. For example, *β*-VAE (Higgins et al., 2017) balances reconstruction and disentanglement, while FactorVAE (Kim and Mnih, 2019) uses total correlation penalties to promote variable independence. Mutual information-based approaches, such as InfoGAN and StyleGAN, enhance disentanglement by maximizing the distinctiveness of latent factors in the output, and supervised or semi-supervised techniques leverage labeled data to guide disentangled representation learning (Kulkarni et al., 2015; Kingma et al., 2014; Kingma and Welling, 2022).

Disentanglement approaches fall broadly into hierarchical and latent space methods. Hierarchical disentanglement organizes the latent space into levels, where higher layers capture abstract features and lower layers focus on specific details. Latent space disentanglement, in contrast, promotes independent variation by assigning each latent dimension to a distinct feature (Burgess et al., 2018; Zheng and Sun, 2019; Watters et al., 2019; Cha and Thiyagalingam, 2023). StyleGAN (Liu et al., 2022; Niu et al., 2023; Wei et al., 2023) achieves this by associating unique features with specific components of a Gaussian latent vector, while hierarchical disentanglement has been applied across domains, including speech (Sun et al., 2020), video sequences (Comas et al., 2021), and multi-modal data (Chen and Zhang, 2023) using attention (Cui et al., 2024), context addition (Li et al., 2021), graph convolution (Bai et al., 2022), and contrastive learning (Xie et al., 2023).

Orthogonal denoising autoencoders (Ye et al., 2016) and factorized latent space models (Jia et al., 2010) enhance disentanglement by learning features from multiple perspectives within a dataset, enabling the integration of diverse data sources. Additionally, correlation loss has been applied to effectively separate identity and expression in facial representations (Sun et al., 2019). Latent feature disentanglement has found applications across various fields, including music (Banar et al., 2023), text (Wang et al., 2022), facial generation (Karras et al., 2019), and protein structure variation (Tatro et al., 2021), though its use in plant phenotyping remains limited.

**Figure 2:**
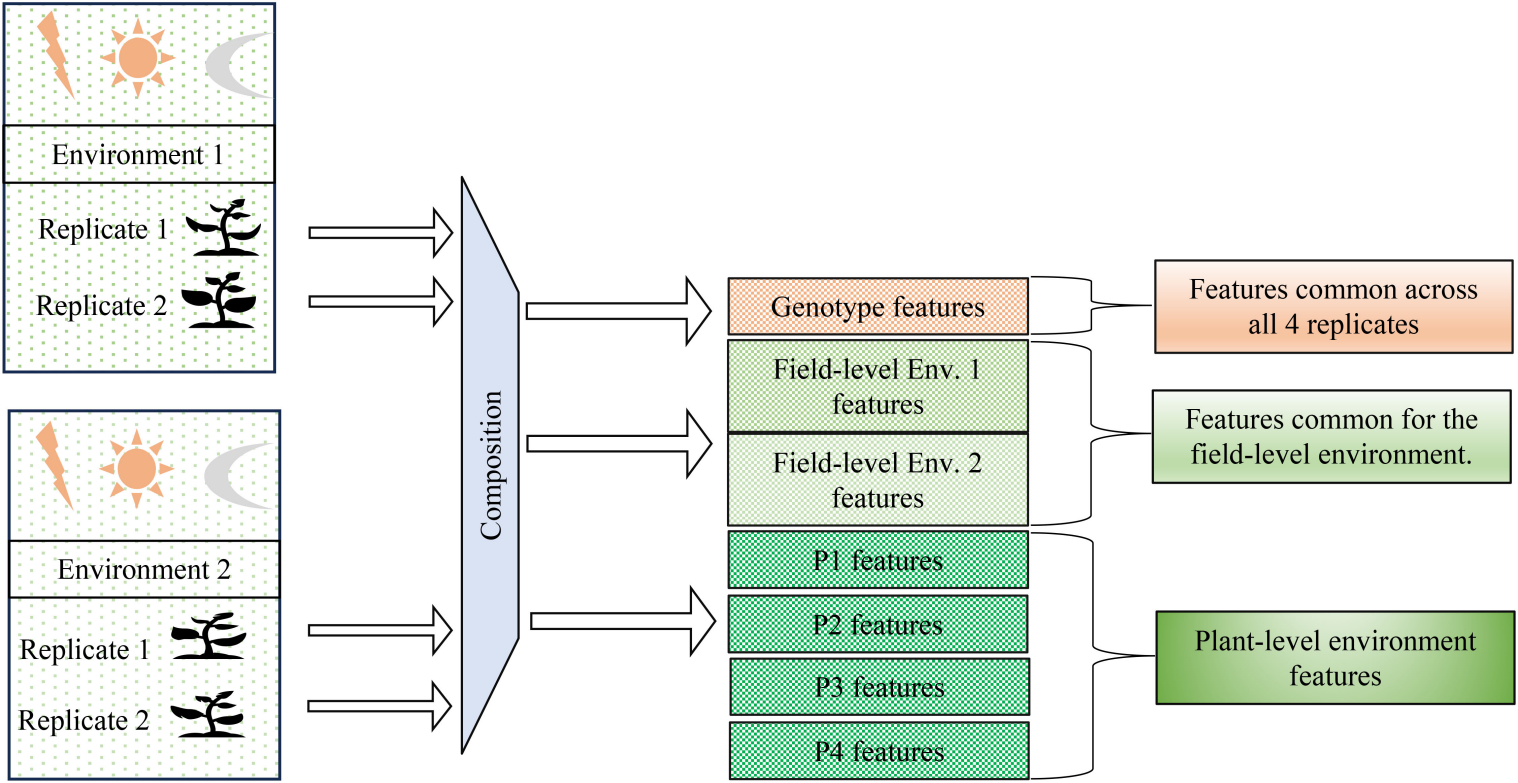
Problem definition: Disentangling genotype-specific, environment-specific, and plant-specific information from hyperspectral data. The goal is to separate features associated with genotype, field-level environmental conditions, and individual plant variations across multiple environments and replicates. This achieved by the method of composition.

In this paper, we propose a compositional autoencoder (CAE), inspired by orthogonal denoising autoencoders (Ye et al., 2016) and factorized latent space models (Jia et al., 2010), to disentangle genotype and environment effects within the latent space. **Figure 2** illustrates the problem definition of the disentangled latent space representation, where environmental factors can include a range of variables such as weather, soil conditions, and treatments applied to plants in a field. Our objectives in this work are as follows:

- Develop a compositional autoencoder (CAE) to separate genotype-specific, macro-, and microenvironmental effects in hyperspectral data.
- Assess whether CAE-generated latent representations improve predictive accuracy for traits like Days to Pollen and Yield.
- Examine the consistency of the CAE’s performance across different model initializations and hyperparameters for potential applications in trait prediction.

## 2 Materials and methods

### 2.1 Equipment and dataset

**Figure 3:**
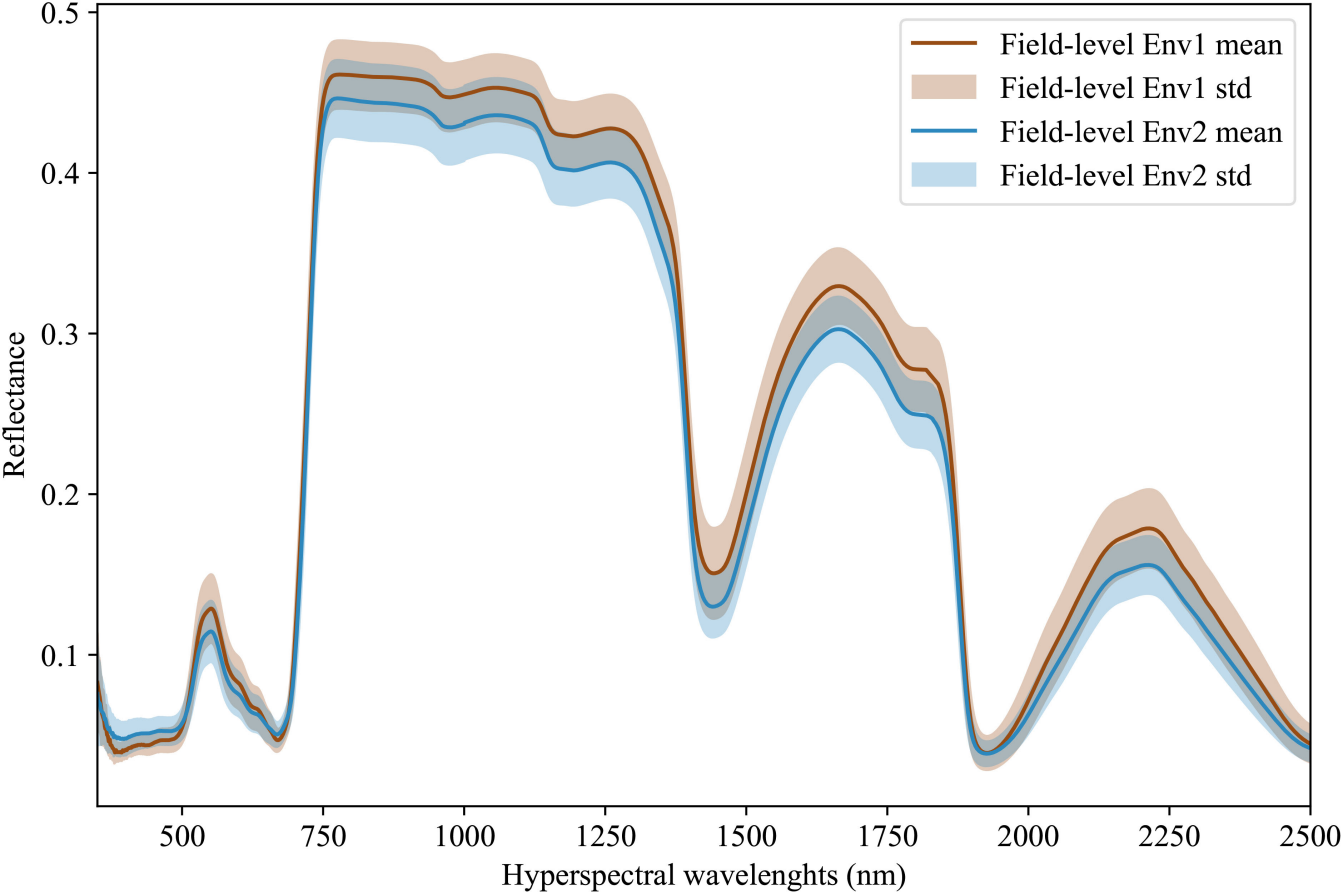
Hyperspectral leaf reflectance data was collected using a FieldSpec4 (Malvern Panalytical Ltd., Formerly Analytical Spectral Devices) with a contact probe. A total of 2151 wavelengths were collected, ranging from 350 nm to 2500 nm. The dataset consists of measurements for a set of 578 different maize inbred genotypes that were grown and phenotyped in two different environments with 2 replicates per environment.


Hyperspectral data is being increasingly adopted by plant scientists as a method to measure or predict plant traits in field and greenhouse settings (Kaleita et al., 2006; Zhang et al., 2023; Yendrek et al., 2016; Tross et al., 2023). For the purposes of this study, we employed data from 578 inbreds, which represent a subset of the Wisconsin Diversity panel (Mazaheri et al., 2019), grown and phenotyped in 2020 and 2021 at the Havelock Farm research facility at the University of Nebraska-Lincoln. In each year, measurements were collected on two replicated plots of each inbred grown in different parts of the field, for a total 2×2×578 = 2312 observed plots. Each plot consisted of two rows of genetically identical plants with approximately 20 plants per row, as previously described in Mural et al. (2022). Hyperspectral data was collected using FieldSpec4 spectroradiometers (Malvern Panalytical Ltd., Formerly Analytical Spectral Devices) with a contact probe. This equipment captures 2151 wavelengths of electromagnetic radiation ranging from 350 nm to 2500 nm. Hyperspectral data was collected from a single fully expanded leaf per plot, selected from a representative plant, avoiding edge plants whenever possible. Three spectral measurements were taken at each of the three points located at the tip, middle, and base of the adaxial side of each leaf. Values were averaged across the nine wavelength scans to generate a final composite spectrum for each plot sampled (Tross et al., 2023). **Figure 3** illustrates the distribution and variability of mean reflectance among the genotypes across two years, which in this paper are referred to as two different environments. We divide the environment into field-level (or macro-environment) and plot-level (or micro-environment) Guil et al. (2009). For the latent features extraction, the data was then normalized using min-max normalization. This normalization is given as:

(1) $$x_{\text{normalized}}=\frac{x-min_{\text{dataset}}}{max_{\text{dataset}}-min_{\text{dataset}}}$$

From the Equation 1, ‘ $min_{dataset}$ ’ and ‘ $max_{dataset}$ ’ are the minimum and maximum values in the entire dataset respectively.

### 2.2 Vanilla autoencoder

**Figure 4:**
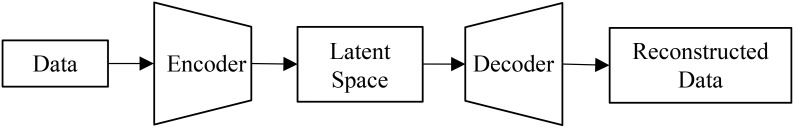
A vanilla autoencoder works to learn a compressed yet highly informative representation of the input data.

We implemented a standard autoencoder (see **Figure 4**) as a baseline for comparison which we refer to below as the ‘vanilla autoencoder’ (AE). Both the encoder and decoder portions of our vanilla autoencoder implementation are made up of multiple fully connected layers stacked together with the non-linear activation function ‘SeLu.’ The encoder encodes the input data (2151 wavelengths) into smaller dimensions (latent space) and decoder works to reconstruct back the original input from this latent space. The **Tables 1**, **2** show the details of each of the layers that constitute the encoder and decoder. For training the vanilla autoencoder, data from each plot in each year is considered as one sample, resulting in a total of 2312 input samples.

**Table 1:** Encoder: Configuration details.

Layer Type	Dimensions	Activation
Linear	input_shape → 2150	SELU
Linear	2150 → 1024	SELU
Linear	1024 → 512	SELU
Linear	512 → zg + ze + zp	None

‘input_shape’ = 1 x 2151, ‘zg’ = dimensions allocated to capture genotype features, ‘ze’ = dimensions allocated to capture macro-environment features, ‘zp’ = dimensions allocated to micro-environment features.

**Table 2:** Decoder: Configuration details.

Layer Type	Dimensions	Activation
Linear	zg + ze + zp → 512	SELU
Linear	512 → 1024	SELU
Linear	1024 → 2150	SELU
Linear	2150 → input_shape	Sigmoid

‘input_shape’ = 1 x 2151, ‘zg’ = dimensions allocated to capture genotype features, ‘ze’ = dimensions allocated to capture macro-environment features, ‘zp’ = dimensions allocated to micro-environment features.

### 2.3 Compositional autoencoder

#### 2.3.1 Architecture

The compositional autoencoder extends the vanilla autoencoder architecture in a way that aims to disentangle the latent space, partitioning the impact of different factors that influence the data into different variables. It consists of an encoder, decoder, and a fusion block. The network operates as follows:

1. Encode Individual Plant Data: The encoder processes data from four plants of the same genotype, compressing it into latent features.
2. Fuse Encoded Data: These encoded representations from all the plants are then fused into a single latent feature.
3. Disentangle Latent Factors: This fused latent feature is then partitioned into three distinct parts: genotype-specific features (common across all plants), macro-environment-specific features (shared by plants from the same environment), and micro-environment-specific features (unique to each plant).
4. Reconstruct Individual Plants: Finally, for each plant, the genotype, macro-environment, and micro-environment features are assembled. This assembled disentangled representation is then decoded to reconstruct the original plant data.

**Figure 5:**
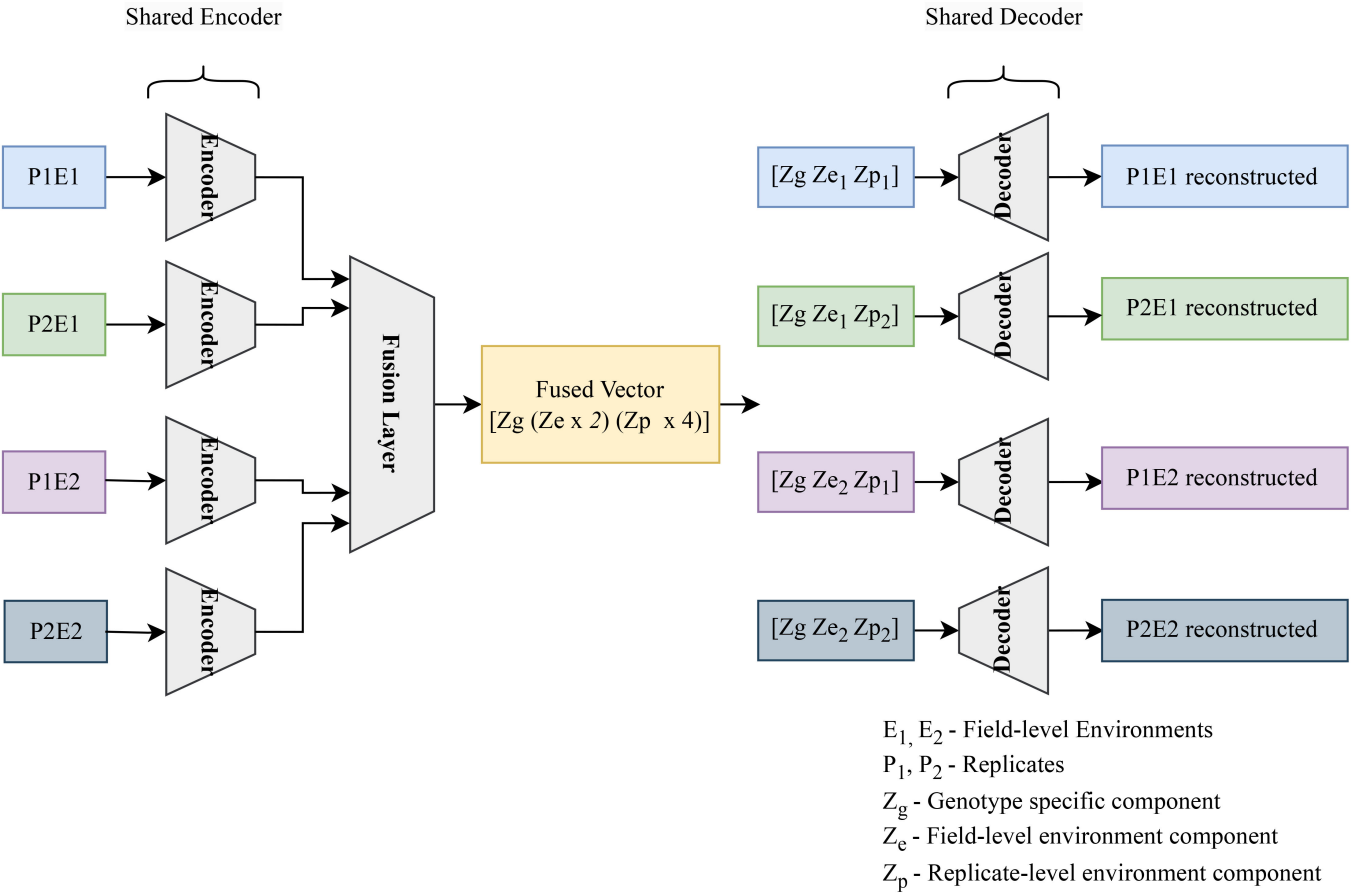
The encoder encodes the hyperspectral data for 4 plants, accounting for a single genotype across two environments (E1, and E2) and 2 replicated per environment (P1E1, P2E1, P1E2, P2E2). The resulting 4 latent vectors are fused using a linear layer. The resulting fused vector contains 3 parts. (1) Genotype representation part. (2) Macro or field-level environment representation part (2 parts to represent E1 and E2 effects). (3) Micro or replicate-specific environment representation component [4 parts to represent each of the plants (P1E1, P2E1, P1E2, P2E2)]. To get the composed encoded form, genotype representation is combined with the field-level environment part and plant-level environment part. These composed encoded vectors are then fed into the decoder to regenerate the original hyperspectral reflectance.

Here, genotype refers to groups of plants with identical genetic makeups, macro-environment refers to common environmental factors experienced by all plants growing in the same field in the same year (e.g. rainfall, temperature), and micro-environment refers to features of the individual replicate growing in the same field within the same environment/year. The table (refer to **Table 3**) illustrates the disentangled latent representation for each plant. A more detailed network architecture can be found in the figure (refer to **Figure 5**). The encoder and decoder used here are the same as vanilla autoencoder with the addition of ‘Fusion’ layer. The layer details are provided in the **Table 4**.

**Table 3:** Disentangled latent-space representation of each plant.

Plant	Representation
Plant 1	{(Zg) genotype, (Ze) macro-environment [1], (Zp) micro-environment [1]}
Plant 2	{(Zg) genotype, (Ze) macro-environment [1], (Zp) micro-environment [2]}
Plant 3	{(Zg) genotype, (Ze) macro-environment [2], (Zp) micro-environment [3]}
Plant 4	{(Zg) genotype, (Ze) macro-environment [2], (Zp) micro-environment [4]}

**Table 4:** Fusion layer details.

Layer Type	Dimensions	Activation
Linear	N(zg + ze + zp) → zg + E(ze) + N(zp)	None

‘N’ = number of replicates per genotype (2), ‘E’ = number of environments. (2), ‘zg’ = dimensions allocated to capture genotype features, ‘ze’ = dimensions allocated to capture macro-environment features, ‘zp’ = dimensions allocated to micro-environment features.

The training process involves dividing the hyperspectral data into groups of four plants (sharing the same genotype). There are a total of 578 such groups (corresponding to the number of genotypes). Each group is fed sequentially through the encoder, resulting in four latent representations. These representations are then fused together. The resulting fused latent space captures three factors: genotype, field-level environment (with two sub-parts for the two environments), and plant-level environment (with four sub-parts for the four plants).

#### 2.3.2 Loss function

We trained the CAE network using a two-part loss function consisting of a reconstruction loss and a correlation loss.

*Reconstruction Loss:* The mean squared error (MSE), was used as the reconstruction loss for the compositional autoencoder. This loss function encourages the network to learn a meaningful disentangled latent space that can be accurately decoded back to the original hyperspectral data.

*Correlation Loss:* A correlation loss was employed to ensure that all parts in the disentangled latent space remain uncorrelated throughout the training process. This loss is defined in Equation 2.


(2) $$\text{Correlation Loss}=\sum_{i=1}^{N}\sum_{j=i}^{N}\left|{\text{CorrMat}}_{ij}\right|-I_{ij}$$

where:

-
${\text{CorrMat}}_{ij}$ represents the correlation coefficient between dimensions *i* and *j* in the latent space.
- *N* is the dimension of the square correlation matrix, which corresponds to the number of dimensions in the latent space.
-
$I_{ij}$ is the identity matrix, ensuring that the diagonal elements (where *i* = *j*) contribute zero to the loss.

The correlation coefficient used here is the Pearson correlation coefficient (*r*), a measure of the linear correlation between two variables. It is calculated using Equation 3.


(3) $$r=\frac{\sum_{i=1}^{n}(p_{i}-\bar{p})\left(k_{i}-\bar{k}\right)}{\sqrt{\sum_{i=1}^{n}{\left(p_{i}-\bar{p}\right)}^{2}\sum_{i=1}^{n}{\left(k_{i}-\bar{k}\right)}^{2}}}$$

where:

- *n* is the number of data points.
-
$p_{i}$ and $k_{i}$ are the elements of the latent space.
-
$\bar{p}$ and $\bar{k}$ are the means of the $p^{th}$ dimension and $k^{th}$ dimension, respectively.

In our case, we aim to achieve zero correlation between the latent space features representing genotype, environment, and individual plant variations. This is enforced by the correlation loss function (Equation 2). This ensures that the disentangled latent space captures these factors independently.

We trained the vanilla autoencoder network using MSE reconstruction loss only.

#### 2.3.3 Training parameters

The data was divided into training and validation with a 85%-15% split. Furthermore, we trained these networks with SGD, Adam, and LBFGS optimizers and found that LBFGS gave us faster convergence (10x). Therefore, all the experiments were carried out using the LBFGS optimizer. The training setup included early stopping criteria, which monitored validation loss and stopped training after it observed no improvements in the metric for 15 epochs.

#### 2.3.4 Parameter tuning for downstream tasks

To improve the performance of latent representations for downstream tasks, we investigated several tuning techniques for both the network and its inputs.

- a) We explored masking a portion of the input data. This technique encourages the model to focus on reconstructing the missing parts, potentially leading to increased robustness and reduced overfitting (Bachmann et al., 2022). We performed a search for the optimal masking percentage.
- b) Considering our dataset size, we conducted a basic architecture search to strike a balance between model complexity and data availability. This helps to mitigate overfitting and improve generalization. We evaluated different network architectures with varying numbers of layers and dimensions in the encoder and decoder.
- c) To ensure the latent representations captured the necessary data complexity, we experimented with different latent space dimensions and their composition of genotype, field-level, and plant-level environmental features.

### 2.4 Downstream tasks performance metrics

To confirm our hypothesis that the disentangled latent representations enhance the latent feature’s ability to predict useful traits, we generated disentangled latent features (disentangled encoded output from the encoder) for all 2312 data points. We then used these features to train models to predict two traits, namely, ‘Days to Pollen’ and ‘Yield (grams)’. We trained several regression models — Random Forests, XGBoost, Ridge Regressions, and PLSR (Partial-Least Square Regression) — to identify a high performing model. We compare the performance of the models trained on the disentangled latent representations from the CAE against the performance of models trained on the latent representations from a vanilla autoencder. The resulting prediction performance was evaluated using an R^2^ metric representing the coefficient of determination. The coefficient of determination, *R* ^2^, is defined as:


(4) $$R^{2}=1-\frac{\sum_{i=1}^{n}{\left(y_{i}-{\hat{y}}_{i}\right)}^{2}}{\sum_{i=1}^{n}{\left(y_{i}-\bar{y}\right)}^{2}}$$

where:

-
$y_{i}$ is the observed value,
-
$\hat{y}$ is the predicted value, and
-
$\bar{y}$ is the mean of the observed data.

## 3 Results and discussion

### 3.1 Disentangled representation from CAE

**Figure 6:**
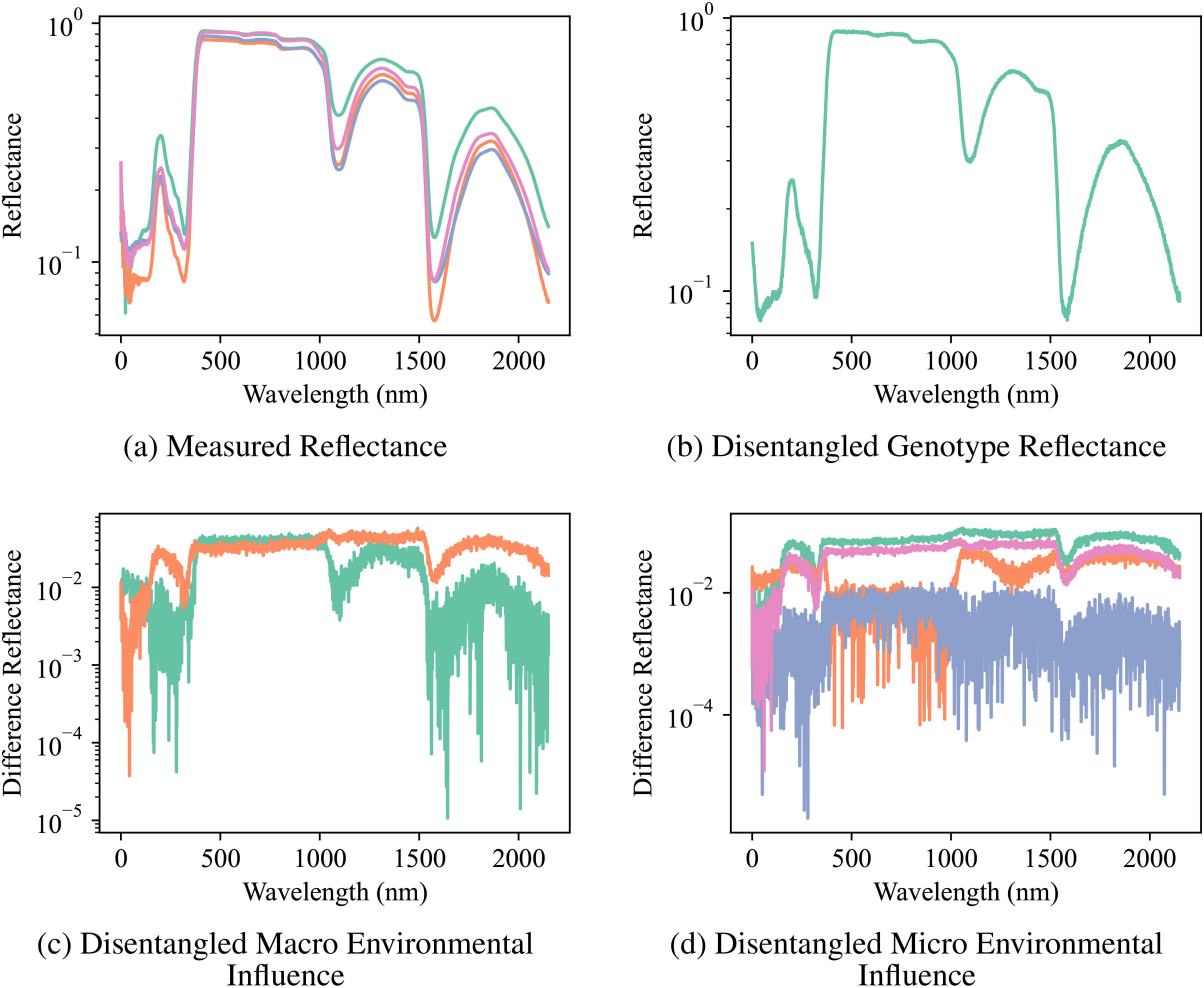
Reflectance Measurements and Disentangled Influences: **(A)** the original measured reflectance spectra for multiple samples of a particular genotype, and the disentangled reflectance components attributed to **(B)** genotype, **(C)** macro-environmental influence, and **(D)** micro-environmental influence. Note the significant variation in the original reflectance due to the combined effects of genotype and environment. Disentanglement enables the visualization of distinct spectral patterns associated with each factor, highlighting the CAE’s ability to separate these influences.

The compositional autoencoder (CAE) successfully disentangled the latent space into genotype, macro-and micro- environmental effects. The **Figure 6** shows a comparison of the original reflectance versus factor-specific (genotype and environments) reflectance. Here, factor-specific reflectance is obtained by modifying the latent space to only keep the effects of either the genotype, or the environments; and subsequently reconstructing the reflectance from them. Therefore, genotype-specific is obtained by replacing the environment components in the latent space with an average of all the environments, and similarly, genotype components are replaced by their average to reconstruct the environment-specific reflectance. **Figure 6B** shows genotype-specific reflectance. As we are focusing on just 1 genotype in this figure, all the replicates will have the same latent space and therefore, the same reflectance. **Figure 6C** shows macro environment-specific reflectance. The distinction between the two macro-environments is visualized by calculating the difference between macro-environment-specific reflectance and genotype-specific reflectance for the two macro-environments. Similarly, **Figure 6D** shows micro-environment-specific reflectance. The visualization shows the difference between genotype-specific reflectance, macro-environment-specific reflectance, and micro-environment-specific reflectance.

**Figure 7:**
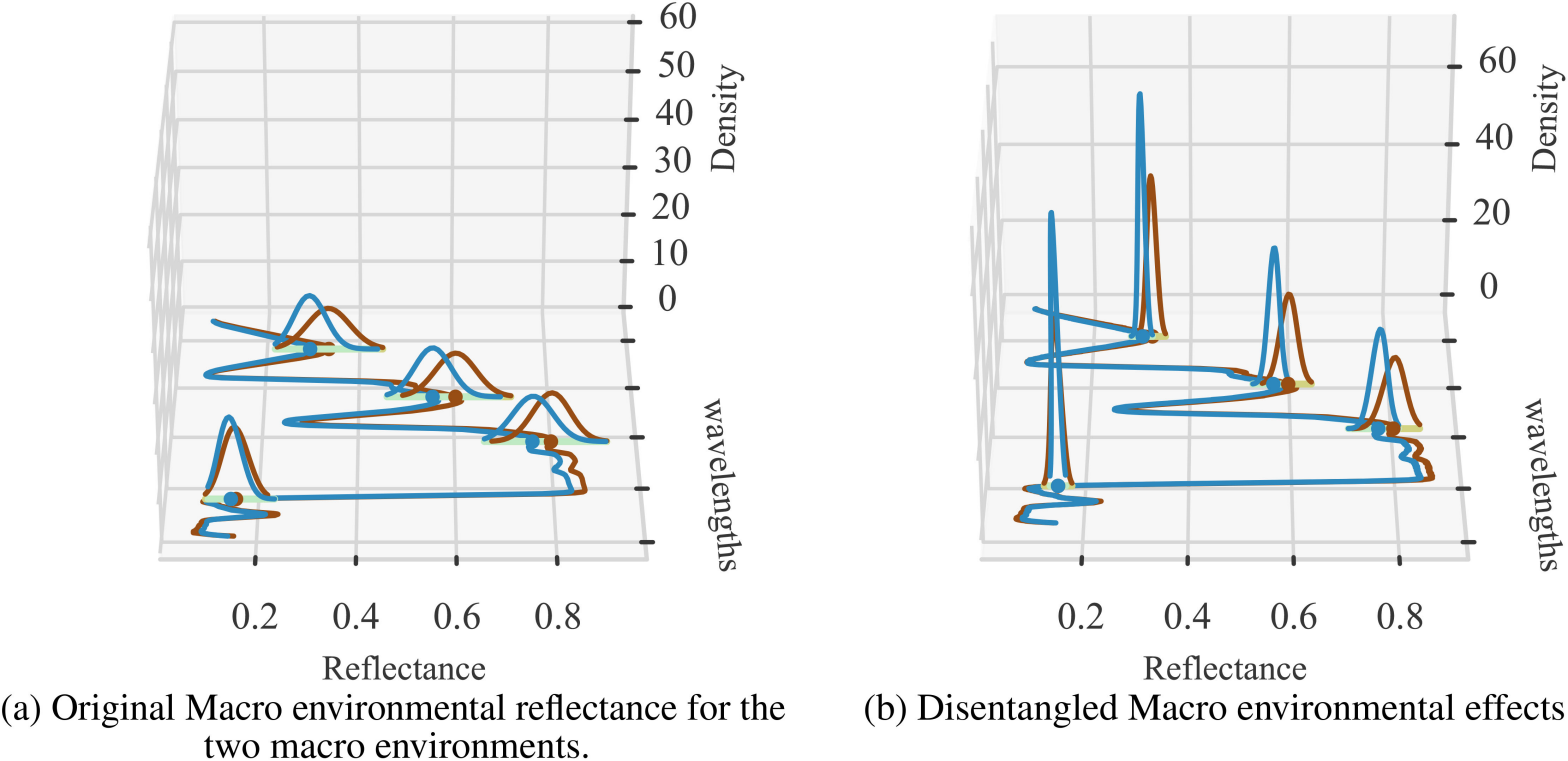
Two sets of visualizations are presented: **(A)** the original environmental effects (Env 1 and Env 2), and **(B)** the disentangled versions. The average KL-divergence observed for the original input data is 0.62, while the disentangled KL-divergence is 2.79. The density distribution of reflectance values is shown at selected wavelengths for clarity, illustrating the separation of environmental factors before and after applying the CAE.

To further verify the degree of environment disentanglement, we calculated the distribution of the two macro environments for the original reflectance (**Figure 7A**) and disentangled environments’ reflectance (**Figure 7B**). A successful disentanglement should yield completely separated distributions. We use KL-divergence to measure the difference between the distributions. We can clearly see that KL-divergence of distributions representing two environments generated from the sensor data is quite low (0.62) while the same for the disentangled reflectance is quite large (2.79). This strongly indicates that the latent representation is, in fact, able to represent the two environments distinctly.

### 3.2 Performance of latent representations on downstream tasks

**Figure 8:**
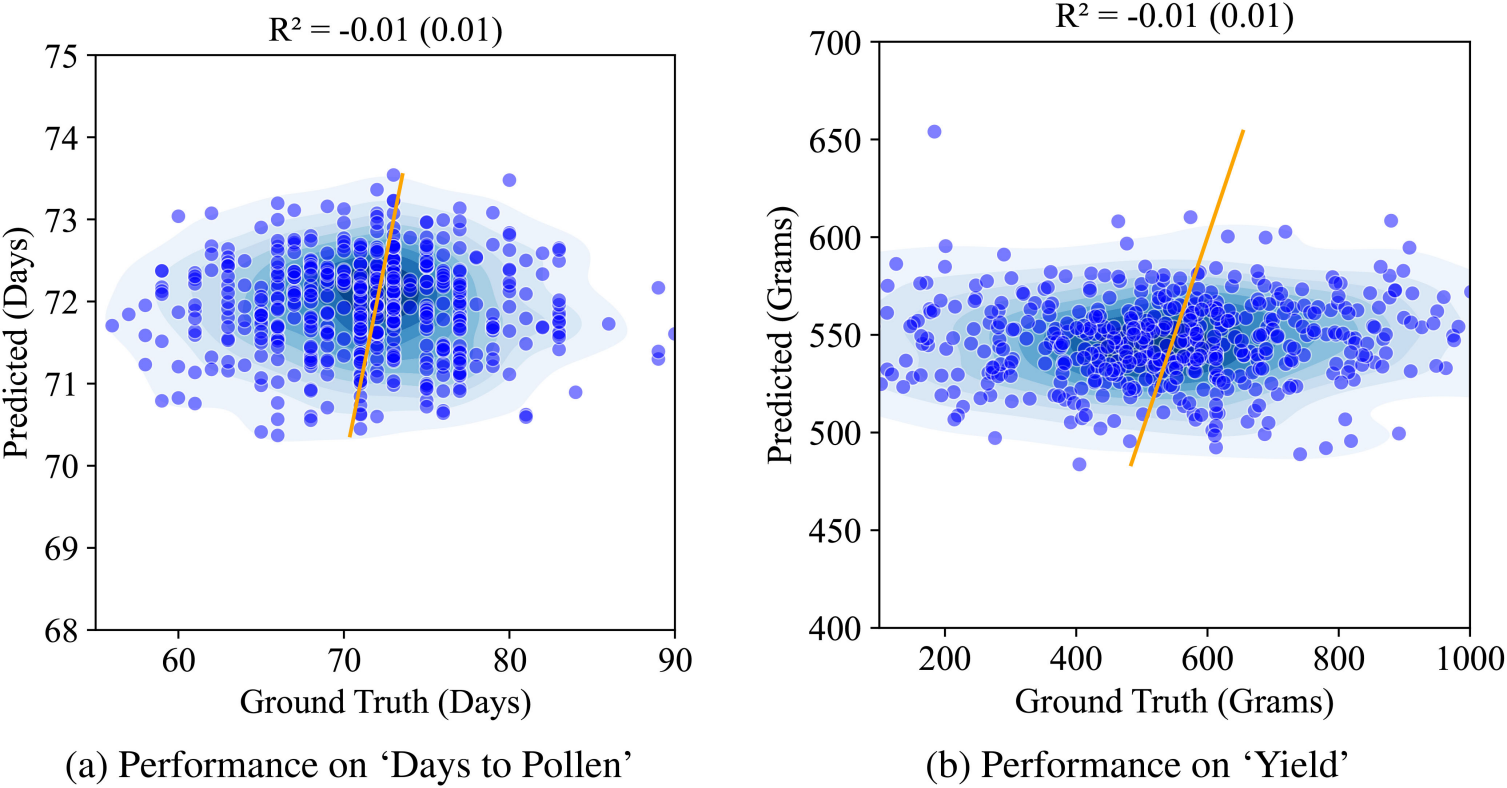
Performance of AE on ‘Days to Pollen’ and ‘Yield’ using Ridge regression: **(A)** Days to Pollen: The x-axis represents ground truth days (60-90 days), and the y-axis represents predicted days (55-90 days). The scatter plot shows points widely scattered, indicating poor prediction accuracy. The model achieves an R of -0.01 (0.01), demonstrating negligible correlation between predicted and actual values. **(B)** Total Grain Mass: The x-axis represents ground truth grain mass (200-1000 grams), and the y-axis represents predicted grain mass (200-1000 grams). The scatter plot shows points widely scattered, indicating poor prediction accuracy. The model achieves an R of -0.01 (0.02), demonstrating negligible correlation between predicted and actual values.

We first report on the performance of our baseline model – the vanilla autoencoder. The latent representation from the vanilla AE was used to train a multiple machine learning models to predict the two traits. We present the Ridge regression model performance here as it yielded the best results among all the models (Random Forests, PLSR, and XgBoost). **Figure 8** shows this performance. We see that the performance for both the traits in question is quite low (*r* ^2^ = 0.01).

**Figure 9:**
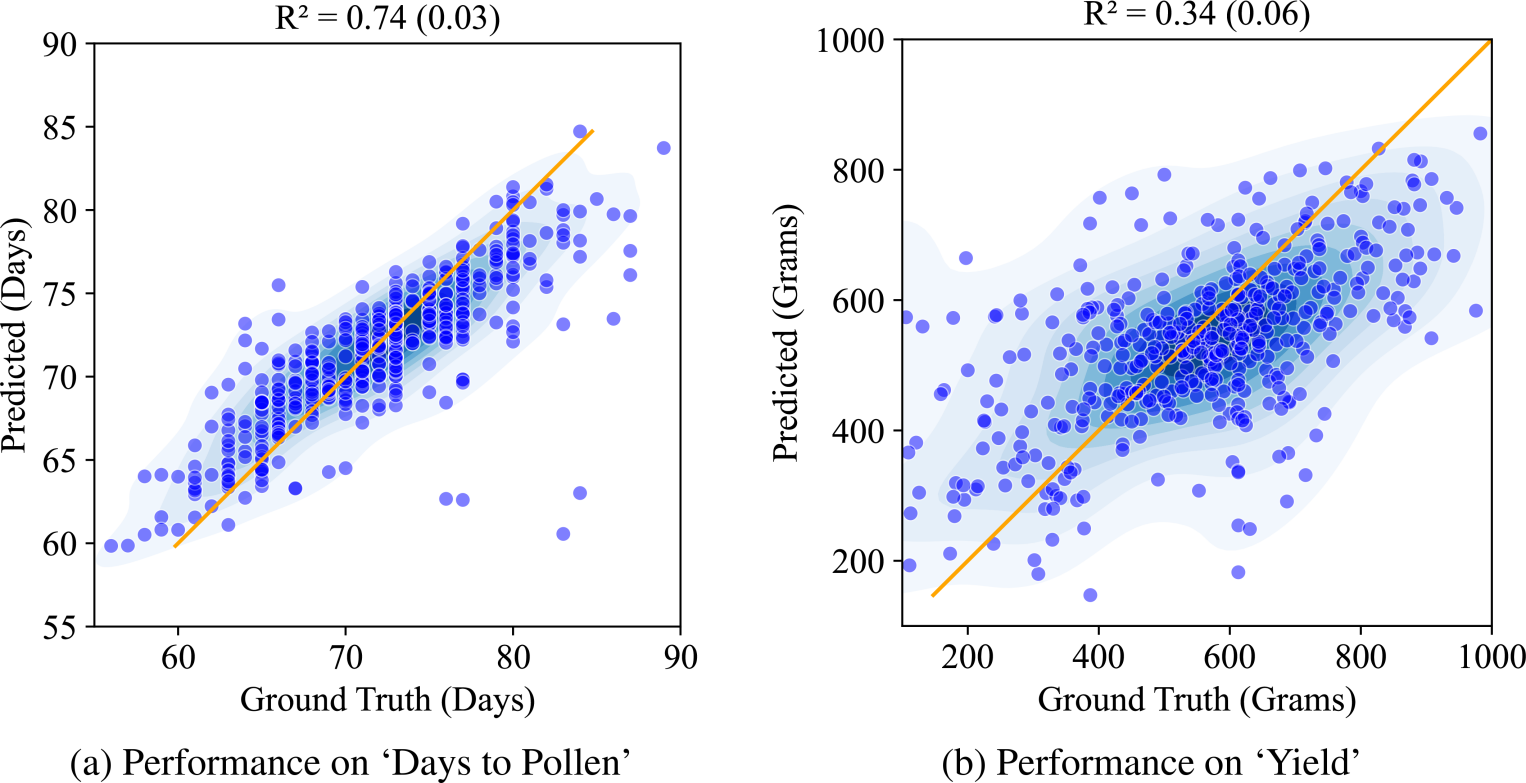
Performance of CAE on ‘Days to Pollen’ and ‘Yield’ using Xg-Boost: **(A)** Days to Pollen: The x-axis indicates ground truth days (60-90 days), and the y-axis indicates predicted days (55-90 days). Points near the line y = x indicate accurate predictions. The model achieves an R of 0.74 (0.03), demonstrating strong prediction accuracy. **(B)** Yield: The x-axis indicates ground truth yield in grain mass (200-1000 grams), and the y-axis indicates predicted grain mass (200-1000 grams). Points near the line y = x indicate accurate predictions. The model achieves an R of 0.34 (0.06), demonstrating moderate prediction accuracy.

Next, we compare this against the performance of the CAE based disentangled representation (similarly trained with multiple machine learning models out of which XgBoost yielded the best results and its performance is reported here). **Figure 9** shows the performance of the structured latent representation generated by the CAE. The Compositional Autoencoder (CAE) performs exceptionally well for the ‘Days to Pollen’ trait, achieving an *r* ^2^ value of 0.74. While its performance in predicting ‘Yield’ is lower, with an *r* ^2^ value of 0.34, this is unsurprising given the complexity of the genetic architecture governing yield. Accurate prediction of yield is inherently challenging due to its intricate genetic influences. Previous studies with these genotypes (Jin et al., 2024) involved costly and labor-intensive genotyping and manual trait measurements. These methods require significant time and effort. Considering these factors, achieving such performance using leaf hyperspectral reflectance collected only at a single time point is significant.

It is worthwhile to compare these results against recent studies based on collecting hyperspectral reflectance measurements of whole canopies instead of the leaf reflectance used here. However, we were unable to find studies reporting results on a diversity panel, so direct comparison is very difficult. The closest was work by Fan et al. (2022), who reported a *r* ^2^ = 0.29 and *r* ^2^ = 0.84 for predicting ‘yield’ and ‘Days to Pollen’, respectively, from hyperspectral imagery of the Genomes2Field project, which consists of around 1000 hybrids. Baio et al. (2023) used hyperspectral images of the canopy of a single commercial hybrid across multiple environments to predict yield with *r* ^2^ = 0.33 with a random forest model. We see that using the CAE approach on leaf scale phenotyping produces competitive results compared to state-of-the-art canopy scale phenotyping. Recent work also suggests that using the hyperspectral data to infer intermediate physiological parameters that are subsequently used to predict yield is a promising approach. For instance, Weber et al. (2012), used leaf reflectance and canopy reflectance to get an *r* ^2^ = 0.7 for leaf reflectance of 100 genotypes. Our findings suggest that CAE-generated latent representations hold promise for capturing relevant yield-related information. Further research is needed to explore the integration of these latent representations with other data sources to potentially improve yield prediction accuracy.

Finally, we compared the effectiveness of using latent representations from (a) a Principal Component Analysis (PCA) on raw data, (b) latent representations from a vanilla autoencoder (AE), and (c) latent representations from a compositional autoencoder (CAE) for predicting the traits of ‘Days to Pollen’ and ‘Yield’. Here, we aim to assess whether the learned latent representations offer benefits compared to using the original data directly.

**Table 5** (yield) and 6 (days to pollen) summarize the performance comparison using the R-squared metric (coefficient of determination) using a 5-fold cross-validation process. The tables showcase the average R-squared values (with standard deviation in parenthesis) achieved by each method and the best-performing machine learning model for that particular scenario. The performances of all the models has been given in the **Supplementary Material** section.

**Table 5:** A final comparison between baseline (PCA on raw data), vanilla autoencoder, and compositional autoencoder for yield prediction.

Metric - Model	Avg. Values	ML Model
*R* ^2^ − *CAE*	0.351 (0.058)	Xg-Boost Regression
*R* ^2^ − *AE*	0.026 (0.017)	Ridge Regression
*R* ^2^ − *PCA*	0.034 (0.016)	Ridge Regression

As observed in **Table 5**, the CAE achieves a significantly higher average R-squared value (0.351) compared to both the AE (0.026) and the baseline using PCA on raw data (0.034) for predicting “Yield.” This suggests that the disentangled latent representations learned by the CAE capture more relevant information for predicting yield compared to the other methods. The best performing model for all three scenarios is Xg-Boost Regression, highlighting its effectiveness for this particular regression task.

Similarly, **Table 6** shows the results for predicting “Days to Pollen.” Here, CAE again demonstrates a clear advantage with an average R-squared value of 0.68, significantly higher than both AE (0.106) and the baseline PCA approach (0.108). This reinforces the notion that the disentangled representations from the CAE do a better job of capturing the factors influencing the number of days to pollen in the data.

**Table 6:** A final comparison between baseline (PCA on raw data), vanilla autoencoder, and compositional autoencoder for Days to Pollen.

Metric - Model	Avg. Values	ML Model
*R* ^2^ − *CAE*	0.68 (0.034)	Xg-Boost Regression
*R* ^2^ − *AE*	-0.01 (0.025)	Ridge Regression
*R* ^2^ − *RAW* − *PCA*	0.108 (0.02)	Ridge Regression
*R* ^2^ − *RAW*	0.16 (0.00)	Ridge Regression

Overall, these results suggest that leveraging the latent representations learned by the CAE offers a substantial advantage for predicting both “Yield” and “Days to Pollen” compared to using the raw data directly or latent representations from the AE. This highlights the effectiveness of disentangled representations in capturing underlying factors that are relevant to these specific traits.

### 3.3 Consistency of latent representations

We evaluate the consistency of the disentangled latent representations by training the model with multiple initial conditions and evaluating its performance across different regression models. This enhances confidence in the reliability and generalizability of the learned latent representations.

The initialization of model parameters can impact the training process and the final performance of the model. Different initializations can lead to the model getting to different local minima, resulting in variable performance. To check the consistency of the performance, we trained both the networks (CAE and vanilla AE) using 4 different initial conditions. By training the model with multiple initial conditions, we can evaluate its robustness and consistency in learning informative latent representations. The **Table 7** (Days to Pollen) and **Table 8** (Yield) show a comparison of performance between a vanilla auto-encoder and compositional autoencoder for the traits of ‘Days to Pollen’ and ‘Yield’ after performing a 5-fold cross-validation. We clearly see the consistency of prediction accuracy across different model initializations.

**Table 7:** Table shows results obtained for Days to Pollen trait using a vanilla autoencoder (AE) and the compositional autoencoder (CAE).

Metric - Model	Init. 1	Init. 2	Init. 3	Init. 4	ML Model
*R* ^2^ **- CAE**	0.681 (0.04)	0.68 (0.035)	0.676 (0.033)	0.68 (0.034)	Xg-Boost Regression
*R* ^2^ **- AE**	0.08 (0.02)	0.127 (0.02)	0.108 (0.03)	0.110 (0.03)	Ridge Regression

The latent vectors generated using these 2 models performed differently with different ML models and the table below shows the best results among all the models that we tested.

**Table 8:** Table shows the results obtained for yield prediction trait using a vanilla autoencoder (AE) and the compositional autoencoder (CAE).

Metric - Model	Init. 1	Init. 2	Init. 3	Init. 4	ML Model
*R* ^2^ **- CAE**	0.351 (0.058)	0.35 (0.054)	0.338 (0.058)	0.345 (0.06)	Xg-Boost Regression
*R* ^2^ **- AE**	0.026 (0.017)	0.027 (0.014)	0.029 (0.015)	0.028 (0.015)	Ridge Regression

The latent vectors generated using these 2 models performed differently with different ML models and the table below shows the best results among all the models that we tested.

We finally report on varying various hyperparameters of the CAE, and their sensitivity to the downstream performance:

- Masking: We evaluated the effect of input masking. Input masking improves the robustness and generalization of autoencoders by forcing them to reconstruct missing or corrupted data, which helps the model learn more significant features and patterns. This technique also acts as a regularization method, preventing overfitting and enhancing performance in various downstream tasks. **Table 9** shows the reconstruction accuracy as a function of masking fraction and suggests that 20% masking is a good choice. We also observed that performance on the downstream task also improved by using masking while training. **Table 10** shows *R* ^2^ observed for different masking percentages.
- Network depth: Network depth is an important hyperparameter to explore because it directly influences the model’s capacity to learn complex patterns and hierarchical representations within the data. Deeper networks can capture more intricate features and dependencies, potentially leading to improved performance on complex tasks, but they also require careful tuning to avoid issues such as vanishing gradients and overfitting. We evaluated how performance varied when the CAE network depth was varied. **Table 11** shows the performance observed for different-sized fully connected networks. We can see that the downstream performance is nearly independent of network depth.
- Size of the latent representation: We next evaluated how the size/dimension of the latent space affected the downstream trait prediction accuracy. Choosing a higher-dimensional latent space can result in better reconstruction accuracy; however, higher-dimensional latent spaces require larger datasets to avoid overfitting of downstream traits. This suggests a balanced approach in designing the dimensionality of the latent space to balance reconstruction accuracy (which improves with increasing latent space dimensionality) with trait regression accuracy (which improves with decreasing latent space dimensionality).

**Table 9:** CAE reconstruction accuracy for different masking %.

Percentage Masking	Val. Loss
0%	0.08
20%	0.05
50%	0.05
70%	0.05

**Table 10:** Downstream trait prediction accuracy (‘Days to Pollen’) for different masking %.

Percentage Masking	*R* ^2^
0%	0.749
20%	0.757
50%	0.756
70%	0.763

**Table 11:** Table shows the performance observed for ‘Days to Pollen’ for different sized networks.

No. Parameters	No. Layers	CAE - *R* ^2^
14.7*M*	4	0.76
5.5*M*	3	0.76
2.2*M*	2	0.76
392*K*	1	0.76

We remind the reader that our disentangled latent space is a vector consisting of three sets of components — ‘Genotype features.’ ‘field-level environment features,’ and ‘plant-level environment features.’ As the genotype is a common characteristic, we assign more dimensions to capture its effects. Field-level environmental features are allocated fewer dimensions, and plant-level environmental features are given the least. **Table 12** shows how the performance of the downstream regression accuracy varies as the latent dimension is doubled from 10 to 20 to 40 to 80 dimensions. We see an asymptotic behavior after a latent space of 20 dimensions.

**Table 12:** Table shows the performance observed for ‘Days to Pollen’ for different latent configurations with 2.2 M training parameters.

Latent space dims (Geno-Env-Plant dims)	CAE - *R* ^2^
10 (6−2−2)	0.69
20 (12−4−4)	0.76
40 (24−8−8)	0.76
80 (48−16−16)	0.77

## 4 Conclusion

This study introduced a novel compositional autoencoder (CAE) framework designed to disentangle genotype-specific and environment-specific features from high-dimensional data, thereby enhancing trait prediction in plant breeding and genetics programs. The CAE effectively separates these intertwined factors by leveraging a hierarchical disentanglement of latent spaces, leading to superior predictive performance for key agricultural traits such as “Days to Pollen” and “Yield.” Our results demonstrate that the CAE outperforms traditional methods, including Principal Component Analysis (PCA) and vanilla autoencoders, in capturing relevant information for trait prediction. The evaluation of various network architectures, latent space dimensions, and hyperparameter tuning further validated the robustness and generalizability of the CAE model. Specifically, the CAE showed consistent performance improvements across different initialization conditions and regression models, underscoring its reliability in practical applications.

By effectively disentangling genotype and environment-specific features, the CAE offers a powerful tool for improving the accuracy and reliability of predictive models in agriculture, ultimately contributing to more informed decision-making in breeding programs and agricultural management. Overall, our contributions in this paper are as follows: a) we report a generalized architecture – compositional autoencoder (CAE) – that can produce a disentangled, low-dimensional, latent representation (that respects hierarchical relationships), given high-dimensional data across a diverse set of plant genotypes. In this case, the effects of genotype and environment on hyperspectral data collected from plants. b) This architecture (CAE) shows an improvement in predicting ‘Days to Pollen’, a measure of flowering time which plays a key role in determining crop variety suitability to different environments, when compared to standard vanilla autoencoder or PCA. c) The CAE latent representation produces models with improved accuracy in predicting the trait ‘Yield’ (i.e. the amount of grain produced by a given crop variety grown on a fixed amount of land), which is both critically important to farmers and considered quite difficult to predict from mid-season sensor measurements when compared to the current state-of-art methods like classical autoencoders.

There are several avenues for future work. First, it will be interesting to explore the viability of compositional autoencoders for making trait predictions using the disentangled GXE features using other sensing modalities Shrestha et al. (2024) like (a) UAV-based hyperspectral imagery and (b) satellite-based multispectral imagery. Second, applying CAE to time-series high-dimensional data collected on diversity panels can produce disentangled low-dimensional time trajectories that could provide biological insight. Finally, integrating these disentangled latent representations with other data (crop models, physiological measurements) may be a promising approach for creating accurate end-of-season trait prediction models using mid-season data.

We conclude by identifying the following limitations of our work: (a) We evaluated the performance of the CAE on two specific traits that were phenotyped in the field experiments. Our future work will focus on evaluating the CAE on a broader range of traits; (b) Our study is based on hyperspectral reflectance data from a specific maize diversity panel. Our future work is focused on extending this to other datasets and environments; (c) While we demonstrate the technical advantages of disentanglement, it is not immediately clear how to connect these disentangled features to biological insights.

## Data Availability

The datasets presented in this study can be found in online repositories. The names of the repository/repositories and accession number(s) can be found below: https://figshare.com/articles/dataset/Hyperspectral_reflectance_data_molecular_and_weights_for_trained_model/24808491/4; https://bitbucket.org/baskargroup/cae_hyperspectral/src/main/.
