# Supplementary material for: Disentangling genotype and environment specific latent features for improved trait prediction using a compositional autoencoder
Source: Front Plant Sci. 2024 Dec 16;15:1476070. doi: 10.3389/fpls.2024.1476070 (PMC11686434; doi:10.3389/fpls.2024.1476070)
Supplement: Supplementary file 1 [file DataSheet1.pdf]

Supplementary Material

1 SUPPLEMENTARY MATERIALS

1.1 Schematic of Problem Statement

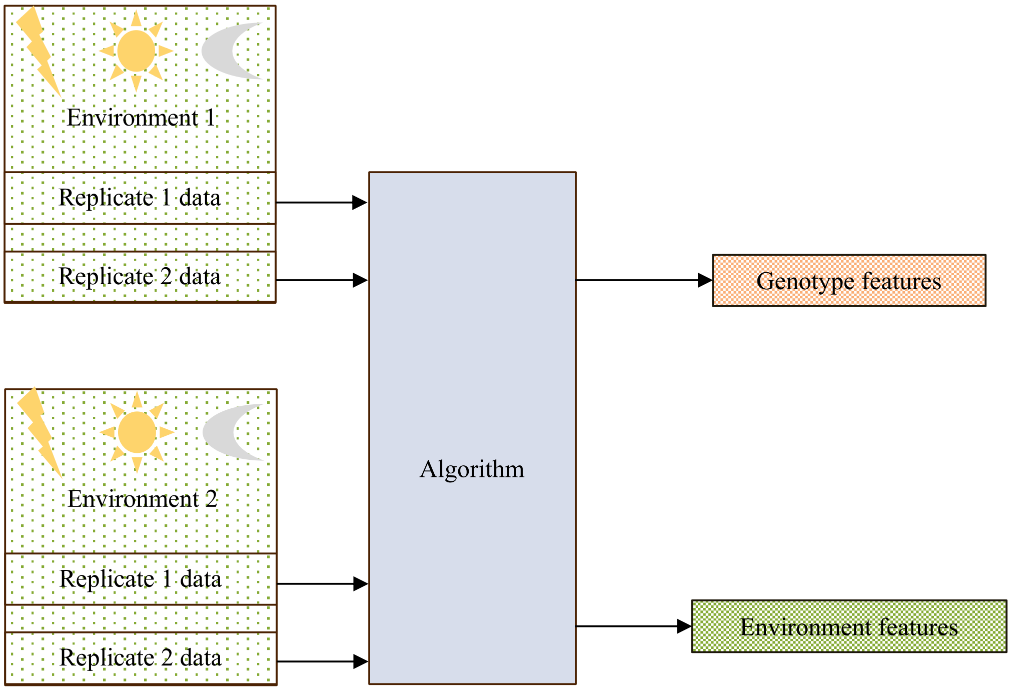

Figure S1. The problem definition: Extract and disentangle the effects of genotype and environment for a given type of sensor data, assuming multiple observations of each genotype in each environment. Our specific test dataset was a set of hyperspectral reflectance data collected from 578 distinct genotypes of maize in two distinct environments, with two replicates of each genotype in each environment (four total replicates per genotype and 1,156 total replicates per environment).

1.2 Data Normalization

We use Min-Max normalization to normalize the data. Figure S2 is the data visualization after normalization.

1.3 Neural Network Training

A neural network is a mathematical model inspired by the structure and function of biological neural networks. It consists of interconnected units or nodes called neurons, organized into layers. The most basic type of neural network is the feedforward neural network, where information moves in only one direction, forward, from the input nodes, through the hidden nodes (if any), and to the output nodes.

Mathematical Representation:

1. **Input Layer:** The input layer consists of input neurons that receive the input features (denoted as $x_1, x_2, \ldots, x_n$).

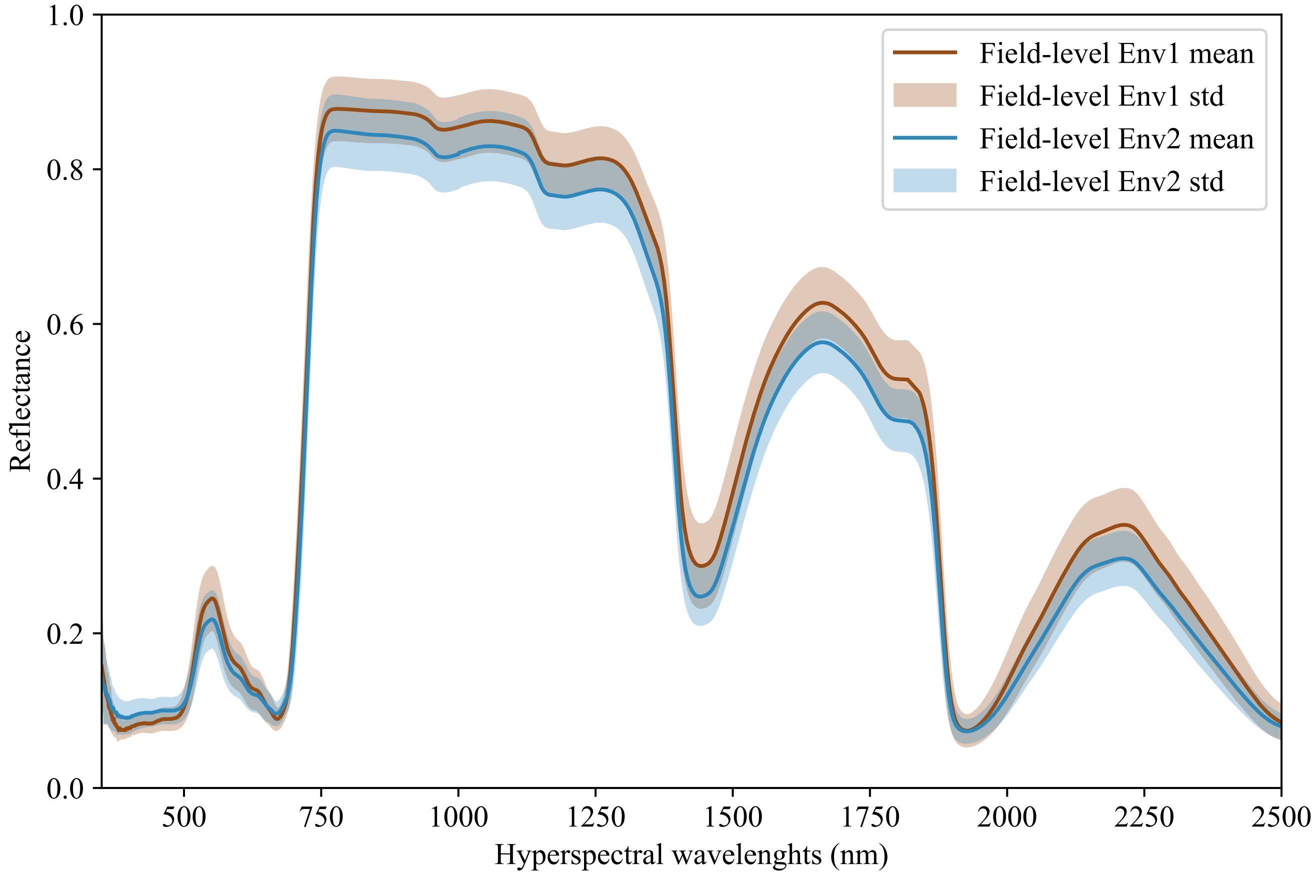

Figure S2. Hyperspectral Data after normalization

2. **Hidden Layers:** Each neuron in the hidden layers applies a weighted sum to its inputs and then passes it through an activation function. The output of the j-th neuron in the k-th layer can be expressed as:

$$a_j^{(k)} = \sigma\left(\sum_{i=1}^{n} w_{ij}^{(k)} x_i + b_j^{(k)}\right)$$

   where $w_{ij}^{(k)}$ is the weight associated with the connection between the i-th neuron in the $(k-1)$-th layer and the j-th neuron in the k-th layer, $b_j^{(k)}$ is the bias term for the j-th neuron in the k-th layer, and σ is the activation function (e.g., sigmoid, ReLU).

3. **Output Layer:** The output layer produces the final output of the network. The process is similar to that of the hidden layers, but the output might pass through a different activation function suitable for the specific task (e.g., softmax for classification).

Training the Neural Network:

The training of a neural network involves adjusting the weights and biases to minimize the difference between the predicted output and the actual output. This process is typically done using the backpropagation algorithm and an optimization technique like gradient descent.

1. **Loss Function:** A loss function (L) measures the difference between the predicted output ($\hat{y}$) and the actual output (y). Common loss functions include mean squared error for regression tasks and cross-entropy loss for classification tasks.
2. **Backpropagation:** This algorithm computes the gradient of the loss function with respect to each weight and bias in the network by applying the chain rule of calculus. It starts from the output layer and propagates the error backward through the network. The gradient of the loss with respect to the weights in layer k is given by:

$$\frac{\partial L}{\partial w_{ij}^{(k)}} = \frac{\partial L}{\partial a_j^{(k)}} \cdot \frac{\partial a_j^{(k)}}{\partial w_{ij}^{(k)}}$$

   where $\frac{\partial L}{\partial a_j^{(k)}}$ is the error propagated from the next layer, and $\frac{\partial a_j^{(k)}}{\partial w_{ij}^{(k)}}$ is the derivative of the activation function with respect to the weights.
3. **Optimizer:** An optimizer uses the gradients calculated by backpropagation to update the weights and biases. The L-BFGS optimizer is a quasi-Newton method that approximates the Hessian matrix to guide the search for the minimum. The weight update equation for L-BFGS can be expressed as:

$$w^{(k+1)} = w^{(k)} - \alpha H_k \nabla L(w^{(k)})$$

   where $w^{(k)}$ are the weights at iteration k, α is the step size, H_k is the approximate inverse Hessian matrix, and $\nabla L(w^{(k)})$ is the gradient of the loss function at iteration k.

This iterative process of forward pass, computing loss, backward pass, and updating weights is continued until the model is sufficiently trained. After training, the neural network can be used for predictions or further analysis.

1.4 Optimizer

1.4.1 LBFGS

To train this neural network, we used the LBFGS optimizer. In the case of a neural network, the optimizer plays a very important role. It drives the weights of the network to a point in the N-dimensional space (N is the number of parameters of the neural network) for which, the loss function for this network is at its minimum. LBFGS is a quasi-newton method of optimization that approximates the hessian instead of calculating it at every iteration to reduce the complexity and time. Newton's method is used to find the minima of a non-quadratic function. It typically starts with a random point and approximates a quadratic around that point and finds the minima for that quadratic and repeats these steps until a minima for the non-quadratic is found. Quadratic is approximated using the 'Taylor series' expansion.

$$g(x) = f(a) + f'(a)(x-a) + \frac{f''(a)}{2!}(x-a)^2 + \frac{f'''(a)}{3!}(x-a)^3 + \cdots \tag{S1}$$

where:

- $f^{(n)}(a)$ denotes the n-th derivative of f evaluated at the point a,
- $n!$ is the factorial of n.

Hessian Approximation in L-BFGS:

L-BFGS maintains a history of the last m updates of the position vectors (s_k) and the gradient vectors (y_k), where k indexes the iteration. The position and gradient vectors are defined as:

$$s_k = w^{(k+1)} - w^{(k)}, \quad y_k = \nabla L(w^{(k+1)}) - \nabla L(w^{(k)})$$

The approximate inverse Hessian matrix (H_k) is updated at each iteration using this history. The update formula is derived from the BFGS update formula but modified to use limited memory. The approximation starts with an initial estimate H_k^0, which is often chosen as a scaled identity matrix:

$$H_k^0 = \gamma_k I$$

where γ_k is a scaling factor that can be computed using different strategies, one common choice being:

$$\gamma_k = \frac{s_{k-1}^\top y_{k-1}}{y_{k-1}^\top y_{k-1}}$$

Then, the approximate inverse Hessian H_k is updated using the formula:

$$H_k = \left(V_k^\top H_k^0 V_k + \rho_k s_k s_k^\top\right)$$

where $V_k = I - \rho_k y_k s_k^\top$ and $\rho_k = \frac{1}{y_k^\top s_k}$.

Step Size Determination in L-BFGS:

The step size (α_k) in L-BFGS is typically determined using a line search method that satisfies the Wolfe conditions. The line search aims to find a step size that ensures a sufficient decrease in the loss function and a sufficient slope of the gradient. The Wolfe conditions are:

1. *Sufficient Decrease Condition (Armijo Condition):*

$$L(w^{(k)} + \alpha_k p_k) \leq L(w^{(k)}) + c_1 \alpha_k \nabla L(w^{(k)})^\top p_k$$

2. *Curvature Condition:*

$$\nabla L(w^{(k)} + \alpha_k p_k)^\top p_k \geq c_2 \nabla L(w^{(k)})^\top p_k$$

where $0 < c_1 < c_2 < 1$ are constants, $p_k = -H_k \nabla L(w^{(k)})$ is the search direction, and α_k is the step size.

The line search algorithm iteratively adjusts α_k until the Wolfe conditions are satisfied, ensuring that the step size leads to a sufficient decrease in the loss function while maintaining the curvature condition.

1.5 Parameter Exploration

Autoencoders are often trained with many different pre-text tasks. A very prevalent method is masking. During the training, the input is partially masked (with zeros) and fed into the Autoencoder, and the reconstruction loss is calculated with respect to the original input data. This forces the network to learn better. The table S1 shows the results with different masking percentages.

Table S1. Table shows the performance observed for different masking percentages. Val. Loss = Coereff Loss + L2 Loss.

Percentage Masking	Val. Loss
0%	0.08
20%	0.05
50%	0.05
70%	0.05

1.6 Downstream Models

1.6.1 Random Forests

Random Forest is an ensemble method composed of numerous decision trees. For regression tasks, it averages the outputs of these trees to obtain the final output, while for classification tasks, it typically uses majority voting. Decision trees recursively split the data into subsets based on attribute tests that maximize information gain. Information gained in each split can be calculated using either entropy or the Gini index. The formula for entropy is $Entropy = -\sum_{i=1}^{n} P(x_i) \log_b P(x_i)$, where b is usually 2 representing binary logarithm. The formula for gini index is $GiniIndex = 1 - \sum {p_i}^2$, where p_i is the probability of class i. The table S2 presents the parameters used for Random Forests in the downstream models.

1.6.2 XGBoost

XGBoost (boosting method) is also an ensemble method. It builds trees sequentially, where each tree tries to correct the errors made by the previous ones. It focuses on boosting weak learners (typically shallow trees) by focusing more on the instances that were misclassified or had higher errors by the earlier trees.

Table S2. Performance Comparison of Models on Days to Pollen (R-squared)

Feature Extraction Model	Prediction Model	Parameters	R-squared
CAE (Ours)	XGBoost	max depth: 15, n estimators: 1500, learning rate: 0.01	**0.76**
	PLSR	10 components	0.02
	Ridge	alpha = 0.001	0.027
	Random Forest	n estimators = 300	0.753
AE	XGBoost	max depth: 1, n estimators: 1500, learning rate: 0.01	-0.01
	PLSR	15 components	-0.012
	Ridge	alpha = 0.001	-0.01
	Random Forest	n estimators = 300	-0.03
Raw Reflectance	Ridge	alpha = 0.05	**0.16**
	PLSR	15 components	0.148
	Random Forest	n estimators = 300	0.08
	XGBoost	max depth: 3, n estimators: 1000, learning rate: 0.01	0.09

1.6.3 Ridge Regression

This works in the same fashion as a typical least square regression where, the algorithm aims the minimize the sum of squared residuals. The change here is the addition of a regularization term that reduces the variance, thereby improving the performance of the model for data that suffers from multicollinearity.

$$J(\beta) = \sum_{i=1}^{n}(y_i - \beta_0 - \sum_{j=1}^{p} X_{ij}\beta_j)^2 + \lambda \sum_{j=1}^{p} \beta_j^2 \tag{S2}$$

where:

- y_i is the observed output,
- β_0 is the intercept term,
- X_{ij} are the predictor variables,
- β_j are the regression coefficients,
- λ is the regularization parameter,
- n is the number of observations, and
- p is the number of predictor variables.
